# Tonsil histopathology in HIV-infected versus HIV-uninfected adults

**DOI:** 10.4102/sajhivmed.v20i1.936

**Published:** 2019-05-28

**Authors:** Ridwaan Essa, Shivesh Maharaj, Kapila Hari, Shahpar Motakef

**Affiliations:** 1 Department of Otorhinolaryngology, University of the Witwatersrand, Johannesburg, South Africa; 2 Department of Internal Medicine, Faculty of Health Sciences, University of the Witwatersrand, Johannesburg, South Africa

**Keywords:** HIV, Malignancy, Tonsil, Tonsillectomy, HIV-infected patients, HIV-uninfected patients

## Abstract

**Background:**

The relationship between HIV and tonsil malignancy has not been fully investigated and established. Both of these diseases prominently feature in the Otorhinolaryngology clinics.

**Objective:**

There is minimal data available on the histopathology of tonsillectomy specimens in the HIV-infected population. This retrospective review compared tonsil histopathology between HIV-infected and HIV-uninfected patients.

**Methods:**

Of the 319 adult patients undergoing tonsillectomy (01 July 2005 to 30 June 2015), HIV results were available for 160. The histological findings were compared in the HIV-infected and HIV-uninfected subgroups. The effects of age, HIV status and CD4 count on the risk of malignancy were determined.

**Results:**

There were 86 patients who were HIV-infected and 74 were uninfected. Reactive lymphoid hyperplasia was the most common diagnosis in both groups (77%). Malignancies were diagnosed in eight HIV-infected and six HIV-uninfected patients, an insignificant difference.

**Conclusion:**

The majority of patients undergoing tonsillectomy had benign conditions. HIV status does not appear to be a specific risk factor for tonsil malignancies, but advanced age may be.

## Introduction

In resource-rich centres, histopathology assessments are performed routinely on adult tonsillectomy specimens.^1^ The merits of routine tonsillar histopathology in poorly resourced centres have been questioned.^2^ A recent systematic review by Rokkjaer et al. (12 studies including 6434 patients) concluded that there is inadequate proof for routine histological examinations from patients who do not exhibit high-risk features.^3^

Beaty et al. in 1998 identified certain features that were associated with an increased risk of tonsillar malignancy:

- History of cancer.
- Tonsillar asymmetry.
- Tonsil firmness.
- Visible lesions.
- Concomitant neck adenopathy.
- Unexpected weight loss.
- Constitutional symptoms (fatigue, night sweats, fever, anorexia).^4^

Recent evidence suggests that HIV infection should be considered an additional risk factor for malignancy.^1^ In view of the limited data in our setting, especially in the adult population where the prevalence of HIV infection is high (18% in the adult population),^5^ we set out to determine whether tonsillar histological studies differed between the HIV-infected and HIV-uninfected patients.

Few studies have documented the effect of HIV infection on tonsillar histology. One Nigerian study by Adoga et al. reviewed 61 patients. The cohort comprised 35 children and 26 adults. They did not comment on the number of patients with HIV infection, although they did detect lymphoma in an adult patient with HIV. They concluded that a request for histopathology on tonsillectomy specimens should be based on the presence of established risk factors with consideration of the cost to patients and to spare histopathological resources.^6^

A South African study by Von Lierop et al. evaluated the impact of HIV on the incidence of adenotonsillar lesions in the paediatric population. Of the 172 patients evaluated in their study, 50% were HIV-positive. They detected only one case of lymphoma, and the patient was HIV-negative. They concluded that, given the high cost of pathological examinations, routine pathology would not be cost effective.^1^

The palatine tonsils play an important role in immunologic surveillance and resistance to infection in the upper aero digestive tract. Palatine tonsils taken from individuals infected with HIV-1 have shown infected lymphocytes localised to the surface of the tonsillar crypt epithelium. Thus, HIV may replicate rapidly at this site because of the numerous T-cells and dendritic cells present. HIV is rarely transferred through the oral cavity and oropharynx as long as the mucosa is intact as demonstrated by a laboratory experiment, whereby minimal transfer of HIV was found after human tonsillar tissue was bathed in HIV semen with an intact mucosa.^7^

HIV infection is associated with an increased risk of a range of malignancies given as follows:

- Acquired immunodeficiency syndrome (AIDS) defining (virus-related):
  ■ Kaposi sarcoma (KS).
  ■ Non-Hodgkin’s lymphoma (NHL).
  ■ Cervical cancer.
- Non-AIDS defining carcinomas (NADC):
  ■ Lung.
  ■ Liver.
  ■ Anal.
  ■ Tonsillar.^8^

In the case of tonsillar malignancies, co-infection with human papilloma virus (HPV) is thought to be responsible for the increase, but HIV itself may play a role.^8^

KS is a frequently reported mesenchymal neoplasm characterised by neoangiogenesis and endothelial-derived, spindle-shaped tumour cells in HIV-infected people, and it is caused by human herpes virus 8 (HHV8). KS is usually isolated to the skin and oral mucosa although it may also occur in the lungs, liver, stomach, bowel and lymph nodes. It has also been described in multiple mucosal sites, including the pharynx, larynx, nasal cavity, oral cavity and the palatine tonsil.^9^

Lymphomas are the most common malignancy in HIV-infected individuals. They represent more than 50% of AIDS-related malignancies. HIV-associated lymphomas are usually:

- high grade
- clinically advanced at presentation
- associated with extra nodal disease.

Common NHLs occurring with HIV are diffuse large B-cell lymphoma and Burkitt’s lymphoma. Hodgkin’s lymphoma does occur, but not as commonly as NHL.^10^

It has been shown that for NADC, the CD4 count does not appear to play a singular role in carcinoma development.^8,11^ It is thought that co-infection with certain viruses resulted in an increased incidence of malignancy. The viruses implicated are HPV, Epstein–Barr virus (EBV), Hepatitis C and HHV 8.^11^ There is also a growing body of evidence that HIV has a direct effect on oncogenes and inactivates tumour-suppressor genes; it may even cause more susceptibility to the effects of the co-existing viral infections. It has been shown that cells involved in the immune system of HIV-positive patients have a shortening of the telomeres.^10^ This may indicate that HIV-positive patients have a more rapid rate of immunologic ageing predisposing to malignancy.^10,11^

## Materials and methods

We reviewed our departmental theatre registries and identified 319 patients with tonsillar histopathology. The surgical procedures were performed between 01 July 2005 and 30 June 2015 at the Chris Hani Baragwanath Hospital, Charlotte Maxeke Johannesburg Academic Hospital and Helen Joseph hospitals, Johannesburg, South Africa. These are teaching hospitals affiliated to the University of the Witwatersrand. The patients’ histology and laboratory records were collected from the National Health Care Laboratory database.

In our cohort, we included adult patients with known HIV serostatus, and 160 patients were identified.

In addition, we recorded other factors, including age, CD4 count and histology result. We did not record clinical data because of incomplete medical records.

## Ethical consideration

Ethics approval was obtained in March 2016 from the University of the Witwatersrand (Ethical Clearance number: M151140).

## Results

There were 160 patients in our cohort. All the statistical tests were carried out with a 5% significance level. All the analyses were carried out using the Statistical Packages for Social Sciences version 1.

### Ages of patients

The ages of the patients in the study range from 18 to 77 years old, with a mean age of 35 years. In the HIV-positive group, the mean age was 32.5 years and the range was 19–66 years. In the HIV-negative group, the mean age was 34 years and the range was 18–77 years.

**FIGURE 1:**
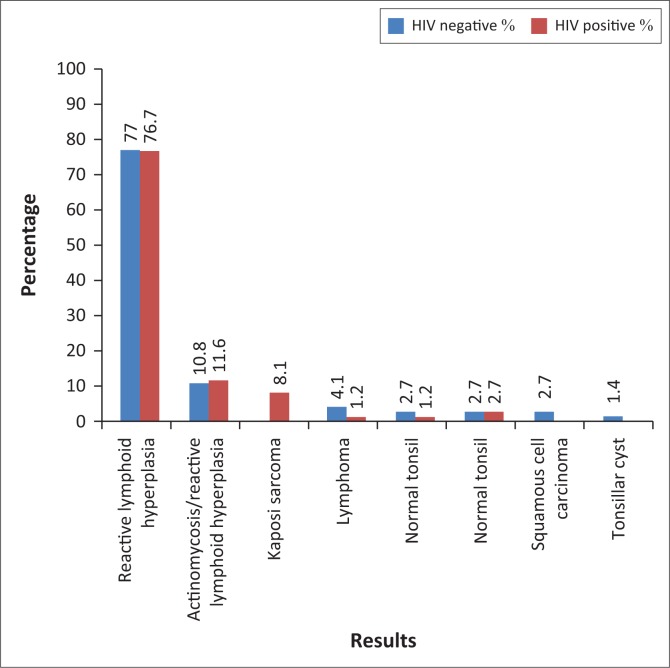
Histology results in HIV-infected (red) and HIV-uninfected patients (blue).

Figure 1 indicates the distribution of tonsillar histopathology results in HIV-infected and HIV-uninfected patients in red and blue colour, respectively. In both groups, reactive lymphoid hyperplasia was found in 77% of patients. Actinomycosis was found in 12% of patients in the HIV-infected group, while in the HIV-uninfected patients, it was observed in 11% of patients.

### Chi-square test of independence

The majority of patients (both HIV-positive and negative) had benign pathology of their tonsils. In the HIV-infected patients, eight patients (9.3%) had a malignant pathology. In the HIV-uninfected group, six patients (8.1%) had a malignant pathology. We concluded that there was no statistical evidence that HIV infection conferred an increased risk for the development of malignant tonsillar disease (see Table 1 and Table 2).

**TABLE 1:** The association between malignancy and HIV status.

Variable	HIV results		Total
	Positive	Negative	
Malignant	8	6	14
Benign	78	68	146

**Total**	**86**	**74**	**160**

Chi-square statistic = 0.071; *p* = 0.790.

**TABLE 2:** Logistic regression of malignancy at age and HIV results.

Variable	Coefficient	Wald statistic	Significance	Odds ratio
Age	−0.053	5.659	0.017	0.948
HIV result (positive)	0.322	0.297	0.586	1.380
Constant	4.264	19.725	0.000	71.106

Logistic regression was performed to test the effects of age and HIV infection on the presence of malignant lesions. The results indicated that when controlling for the effect of age in the model, HIV-infected patients are 1.380 times more likely to have malignant tonsillar pathology than HIV-uninfected patients. The Wald tests showed that only age significantly predicted the presence of malignant pathology (*p* = 0.017). HIV infection did not influence the risk of developing malignant pathology (*p* = 0.586). This result is in accordance with the chi-square test in Table 3.

**TABLE 3:** CD4 count: Effect of CD4 count on malignant lesions.

Variable	CD4 count (cells/mm^3^)		Total
	≤ 200	➢ 200	
Malignant lesion	2	5	7
Benign lesion	27	73	100

**Total**	**29**	**78**	**107**

Chi square = 0.008; *p* = 0.928.

Patients with a lower CD4 count (< 200 cells/mm^3^) would be expected to have more severe HIV disease and, therefore, a greater chance of having an HIV-associated malignancy. In our study, a low CD4 count did not appear to affect the occurrence of malignant lesions (*p* = 0.928).

## Discussion

We reviewed 160 tonsillectomy specimens. The most common finding on histopathology was reactive lymphoid hyperplasia, which is a pattern of hyperplasia seen in tonsils secondary to an inflammatory or immune response.^12^ This was present in 123 patients (77%). The findings were the same in both the HIV-infected and HIV-uninfected groups. Our findings are consistent with previously published studies, which found reactive lymphoid hyperplasia in the vast majority of patients.^13,14^

There are minimal data available on the histopathology of routine tonsillar specimens in the adult HIV-infected population. A South African study by Lierop et al. reviewed the tonsil histology in 344 paediatric patients.^1^ Reactive lymphoid hyperplasia was present in all their patients with HIV infection (four patients). Our findings suggest that reactive lymphoid hyperplasia remains the most common finding in patients undergoing tonsillectomy, regardless of HIV status or age.

Dell’Aringa et al. studied 250 patients between the ages of 2 and 34 years; the mean age was 7.3 years. Of these, 245 (95%) had lymphoid hyperplasia or inflammation; 2 (0.8%) had tonsillar cysts and 2 (0.8%) had actinomycosis infection.^15^ Our study, which was of a similar size, had an older population; the mean age was 35 years. We had greater proportions of patients with actinomycosis 45 (14%), squamous cell carcinoma (SCC) 8(2.5), KS 7 (2.2%) and lymphoma 4 (1.3%) in our study. In both studies, granulomatous disease was present in only one patient.

Actinomyces are commonly found in the oral cavity where they are commensals. Their role in the development of tonsillar disease has not been firmly established.^16,17^ In our study, actinomycosis was equally present in both patients with HIV (11.6%) and without HIV (10.8%). The presence of actinomycosis infection in tonsillectomy specimens is well described. Rebechi et al. evaluated the routine histopathology of 281 patients who underwent tonsillectomy, most of whom had recurrent bouts of infection. They found evidence of chronic tonsillitis with colonies of actinomyces in 9.6% of their patients.^18^ In light of our findings, we concur with Hasan et al., who suggested that actinomycosis is likely to have a causal association with recurrent tonsillitis and tonsillar hypertrophy.^17^

South Africa has one of the highest burdens of TB in the world, with an estimated 450 000 active cases in 2013.^19^ We, however, did not detect any evidence of TB in the specimens that we reviewed. This is not unexpected as tonsillar involvement in TB infection is rare. Ricciardello et al. looked at otorhinolaryngology-related TB in 304 patients. Their study was conducted in Naples. They found tonsillar involvement in only two patients.^20^

SCC is the most common malignancy in patients with non-benign tonsillar lesions. It accounts for up to 7% of malignancies. Courville et al. tested 1093 adult patients, and of these, 75 (7%) had SCC.^21^ Malignancies were suspected prior to the confirmatory histology in all of their patients. Four of our patients had SCC on pathological evaluation; three patients were HIV-uninfected and one patient had HIV infection. Randall et al. found 22 patients with SCC (0.04%). The relatively low overall prevalence in their study was probably because of the high number of paediatric tonsillectomy specimens (96%). In their adult population, SCC was present in 25 patients (1.2%).^22^

Lymphomatous lesions are well described in patients with tonsillar malignancy. Their occurrence ranges from 0% to 1.74%.^1,13,14,15,21,22^ Ikram et al. studied 200 patients between the ages of 4 and 49 years. They detected lymphoma in only one patient (0.5%).^13^ Younis et al. evaluated the histopathology in 2438 routine tonsillectomy specimens. In the 339 adult specimens they reviewed, they detected lymphoma in six patients (1.74%). All their lymphoma patients had suspicious clinical findings preoperatively.^14^ We had four patients (1.3%) with lymphoma; one of these patients had concomitant HIV infection.

The relationship between tonsillar malignancies and underlying HIV is not well established. We found 14 patients with malignancies, and of those patients, eight were HIV-infected and six were HIV-uninfected. The results showed no significant correlation between tonsil malignancy and HIV infection.

HIV infection is associated with an increased risk of a range of cancers, including KS, NHL and cervical cancer, which are considered virus-related and AIDS-defining diseases. The spectrum and incidence of NADC (smoking- and virus-related) has also been shown to be augmented. Franzetti et al. found a higher than expected incidence of tonsil carcinoma in their review of 5924 HIV-infected patients.^8^ Mitsuyasu reviewed the incidence of both AIDS-defining and NADC in the United States. He did not find an increased incidence of tonsillar malignancies.^11^ Grulich et al. performed a meta-analysis of people with HIV/AIDS (444 172 patients). They found an increased incidence of oral cavity and pharyngeal tumours. Unfortunately, they did not specify whether any of their patients had tonsillar malignancies.^23^

In our study, KS was detected in seven patients, all of whom were HIV-infected. There is an established strong correlation between HIV and KS, which has been borne out of the literature.^8,11,24,25^ HIV-infected individuals in southern Africa have a higher risk of developing KS than their counterparts in Europe.^24^ Prior to the HIV epidemic, the prevalence of KS was low in Africa. There has been a marked surge in the number of KS cases. HHV8 is implicated in this resurgence of KS. The mechanism of how HHV8 causes KS is not completely understood, but it is believed to be instigated by oncoprotein production and the inhibition of tumour-suppressor genes. It usually begins in the head and neck. This may be because of the fact that the oropharynx is the main reservoir for HHV8.^25^

Franzetti et al. found a significant association between NADC and low CD4 counts (< 200 cells/mm^3^). They evaluated 5924 HIV-infected patients over a 26-year period. They used regression models to compare the cancer risk in their HIV-infected patients to age- and gender-matched individuals.^8^ We could not confirm this relationship. We cannot exclude the possibility that the retrospective nature of our study, the relatively small number of events and the large deviations of CD4 counts would make our results less reliable. Advancing age was the only predictor of malignancy in both the HIV-infected and HIV-uninfected subgroups.

### Study limitations

The size of our study cohorts may be adequate, but is not ideal for a statistically powerful analysis. Unfortunately, many patients were excluded because of the absence of HIV results.

The lack of clinical data on the patients is not ideal as we were unable to establish the absence or presence of established high-risk features.

The significance of our study lies in the findings that advancing age may be an additional risk factor to consider, and HIV status does not confer an added risk in the development of tonsillar malignancies.

## Conclusion

Our study findings are consistent with current recommendations that there is inadequate proof for routine histological examinations from patients who do not exhibit high-risk features.^3^ Previously identified features that carry an increased risk for malignancy include history of cancer, tonsil firmness, visible lesions, concomitant neck adenopathy, unexpected weight loss and constitutional symptoms (fatigue, night sweats, fever and anorexia).^4^ The additional risk factor that we identified was an advanced age; this needs to be further by larger studies.

Tonsil histology should probably be reserved for patients with clinically suspicious lesions independent of HIV status.

## Summary

- There is minimal data on the histopathology of tonsillectomy specimens in the HIV-infected population.
- This retrospective review compared tonsil histopathology between HIV-infected and HIV-uninfected patients.
- There were 86 patients HIV-infected patients and 74 HIV-uninfected patients in this study.
- Reactive lymphoid hyperplasia was the most common diagnosis in both groups (77%).
- Malignancies were diagnosed in eight HIV-infected and six HIV-uninfected patients, an insignificant difference.
- The majority of patients undergoing tonsillectomy had benign conditions.
- HIV status does not appear to be a specific risk factor for tonsil malignancies, but advanced age may be.
